# Comparison of light-induced formation of reactive oxygen species and the membrane destruction of two mesoporphyrin derivatives in liposomes

**DOI:** 10.1038/s41598-019-47841-x

**Published:** 2019-08-05

**Authors:** Barnabás Bőcskei-Antal, Ádám Zolcsák, Nikoletta Kósa, István Voszka, Gabriella Csík, Katalin Tóth, Levente Herenyi

**Affiliations:** 1 Department of Biophysics and Radiation Biology, Semmelweis University, Budapest, Hungary; 2 Biophysics of Macromolecules, DKFZ, Heidelberg, Germany

**Keywords:** Biophysics, Membrane biophysics

## Abstract

The photodynamic effect requires the simultaneous presence of light, photosensitizer (PS) and molecular oxygen. In this process, the photoinduced damage of cells is caused by reactive oxygen species (ROS). Besides DNA, the other target of ROS is the membranes, separating internal compartments in living cells. Hence, the ability of ROS formation of porphyrins as PSs, in liposomes as simple models of cellular membranes is of outstanding interest. Earlier we compared the binding parameters and locations of mesoporphyrin IX dihydrochloride (MPCl) and mesoporphyrin IX dimethyl ester (MPE), in small unilamellar vesicles (SUV) made from various saturated phosphatidylcholines. In this study, we used the same kinds of samples for comparing the ROS forming ability. Triiodide production from potassium iodide because of light-induced ROS in the presence of molybdate catalyst was applied, and the amount of product was quantitatively followed by optical spectrometry. Furthermore, we demonstrated and carefully studied SUVs disruption as direct evidence of membrane destruction by the methods of dynamic light scattering (DLS) and fluorescence correlation spectroscopy (FCS), applying unsaturated phosphatidylcholines as membrane components. Although the ROS forming ability is more pronounced in the case of MPCl, we found that the measured disruption was more effective in the samples containing MPE.

## Introduction

Photodynamic therapy (PDT) is a form of phototherapy, which involves a dye with negligible dark toxicity called the photosensitizer (PS), the appropriate wavelength light to excite the PS, and tissue oxygen to elicit the death of the desired cells. PDT has been used as a treatment modality for tumorous diseases since the early seventies. Accordingly, it has been utilized in a wide spectrum of the tumorous conditions such as bladder cancer, head and neck cancer, breast cancer, non-small cell lung cancer and some forms of melanomas. Since then, PDT became a potential therapeutic modality for other nonmalignant diseases such as age-related macular degeneration (AMD), psoriasis, atherosclerosis, and it also became an antimicrobial method of great promise. The concept itself proved to be useful in various other fields as well, including sterilization of drinking water or blood products, or to be used as a herbicide or an insecticide in agricultural application^1–5^.

The photochemical mechanism, which lies in the background of PDT, is that a PS is excited by a specific wavelength light and then the excited triplet state of the PS can interact with the ground state oxygen (3Σg) in the tissue or with other substrates forming singlet oxygen (1Δg) and other reactive oxygen species (ROS). The light activation allows the treatment to have several advantages compared to other modalities. PDT is considered as a relatively non-invasive, localizable process. It can be combined with other treatment options as well. Furthermore, thanks to its numerous cellular targets, it is thought that drug resistance is not a concern in the case of PDT. The selective accumulation of PSs in tumorous tissue is a pivotal question for the effectiveness of the therapy. Moreover, the subcellular location of the PS is of crucial importance in determining the therapeutic efficacy of PDT^6^.

Most of the PSs are located in the membranes, and they can have significant differences, depending on their binding site in the membrane; since the deeper the PS is located, the more accessible it is to the 3–4 times higher oxygen concentration in the membranes compared to the cytosol. Another crucial question is the structure and lipid composition of membranes. A possible consequence of the presence of light-induced ROS is the formation of damage in the membranes which may result in the leakage of ions and molecules through the membrane^2,4,7–12^.

Recently, it has been shown the information that the presence of ROS in the plasma membrane can lead to the effective disruption of the membrane^13,14^.

Earlier in the course of our research, we determined the assumable location of two different porphyrin PSs, namely mesoporphyrin IX dihydrochloride (MPCl) and mesoporphyrin IX dimethyl ester (MPE), in liposomes as simple models of cellular membranes by fluorescence line narrowing spectroscopy. Liposomes were small unilamellar vesicles (SUV) made from various saturated phosphatidylcholines with different length of hydrocarbon chains (14, 16, 18 carbon atoms for each, respectively), namely, dimyristoyl-, dipalmitoyl-, and distearoyl-phosphatidylcholine (DMPC, DPPC, DSPC respectively). We also compared the binding parameters of these porphyrins in the same SUVs^15,16^.

In the next step of our project, we investigated the ROS formation in our model systems and then to find direct evidence of the membrane-damaging effect. In this article, we determined the ROS forming ability due to white light irradiation in different model systems. For the detection of ROS we used iodide with molybdate catalyst:

1 $${\rm{ROS}}+{{\rm{3I}}}^{-}+{{\rm{2H}}}^{+}\to \frac{{{(\mathrm{NH}}_{{\rm{4}}})}_{{\rm{2}}}{{\rm{MoO}}}_{{\rm{4}}}}{{\rm{catalyst}}}\to {{{\rm{I}}}_{3}}^{-}+{{\rm{H}}}_{{\rm{2}}}{\rm{O}}{\rm{.}}$$

This method is accepted to be a sensitive probe for ROS formation and its major advantage is that the produced triiodide is easily detectable with optical spectrometry^17–19^.

We learned that the presence of ROS in the cellular environment is necessary, but not a sufficient condition for damage. Furthermore, the leakage of ions through membranes could be a possible concomitant of damage^4,9,20^. However, we wanted to obtain unambiguous evidence for the damaging effect. Therefore, we intended to demonstrate the membrane disrupting abilities of ROS following the change of mobility of the SUVs by dynamic light scattering (DLS) to gain further insight into the membrane-damaging capabilities of ROS. It is known that ROS readily oxidizes the double bond of unsaturated hydrocarbon chains of fatty acids, but for comparison, we were also interested in what kind of changes could happen due to photoreaction in the case of membranes made from saturated phosphatidylcholines. Since the MPCl seemed more efficient in ROS formation we used the MPCl-DPPC SUV sample for the first experiment, but it was not sensitive for the irradiation: significant changes in the size distribution functions were not observable. Then we prepared another sample, applying unsaturated phosphatidylcholines as membrane components. The new membrane model consisted of a mixture of DPPC and dioleoyl-phosphatidylcholine (DOPC), but we could not observe light-induced detectable changes in the size distribution of this modified sample either.

After these negative results, we checked our idea with direct addition of H_2_O_2_ as a ROS to both types of SUVs and compared the membrane disrupting capabilities. We again studied the size distribution of SUVs by DLS. While the DPPC sample more or less remained unchanged after the ROS addition, the composite sample showed remarkable changes.

For that reason, we turned to a more sensitive method of fluorescence correlation spectroscopy (FCS). We had to prepare a modified sample for this measurement, but finally, we saw the disrupting effect due to ROS formation by white light irradiation. When we applied MPE as PS with the less efficient ROS forming ability, we could observe more effective disruption, measured by the original DLS method. Finally, we could demonstrate that this seemingly surprising result is in accordance with the literature and with our earlier results.

## Methods

Samples were prepared according to methods described in our previous articles^15,16^, but in this study, preparation was partly modified for our current aims.

### Chemicals

The applied photosensitizers (PSs) were mesoporphyrin IX dihydrochloride (MPCl) and mesoporphyrin IX-dimethyl ester (MPE) (Frontier Scientific, Inc., Logan, UT). Their stock solutions were prepared in dimethyl-formamide (DMF) (Sigma, St. Louis, MO) with a concentration of around 2 mM and kept in a dark place (under 5 °C degree) to avoid photodegradation. Liposomes were made from saturated lipids as dimyristoyl-, dipalmitoyl- and distearoyl-phosphatidylcholine (DMPC, DPPC, DSPC) and unsaturated lipid as dioleoyl-phosphatidylcholine (DOPC) (Sigma, St. Louis, MO). The applied fluorescent dye for fluorescence correlation spectroscopy (FCS) was 3,3′-Dioctadecyloxacarbocyanine Perchlorate (DiO) (Sigma, St. Louis, MO).

### Sample preparation

We prepared monocomponent small unilamellar vesicles (SUVs) from DMPC, DPPC and DSPC, and multicomponent SUVs from a mixture of DPPC + DOPC in 80:20% and 70:30% (m/m). As a first step, we prepared a thin lipid layer on the wall of a glass vial. 10 mg of the phosphatidylcholines were dissolved in 200 μl chloroform and then dried with nitrogen stream. Afterward, it was kept in a desiccator at least overnight. Monocomponent lipid films were hydrated with phosphate buffered saline (PBS) (10 mM phosphate, 137 mM NaCl, pH 7.4) at a temperature just above the main (liquid-gel) transition temperature (T_m_) of the corresponding lipid membrane (≈24, 42, and 55 °C for DMPC, DPPC, and DSPC, respectively). The DPPC + DOPC samples were hydrated with PBS over 42 °C. After this procedure, we applied two different methods for SUV preparation:

1. Samples were sonicated for 10 min twice (MSE Soniprep 150 Ultrasonic Disintegrator, frequency 23 kHz; wave-amplitude 8 μm) with a 5 min pause to avoid overheating. Remnants of multilamellar vesicles and contaminants were removed by centrifugation (Beckman J2-21 centrifuge, 15000 rpm, 10 min).
2. In this case, we used a thermostated (45 °C) extruder (Avanti Polar Lipids, Inc., Mini-Extruder). In the first step, the lipid suspensions were forced 15 times through a polycarbonate filter with 400 nm pore sizes. After that, we used a 100 nm pore size with 15 extrusions. In the last extrusion step, the samples were pressed through 50 nm pores 35 times.

Based on repetitive dynamic light scattering (DLS) measurements in both cases, we got well reproducible SUV samples with narrow size distribution (with different typical sizes), but the second method provided samples with higher stability in time. The final phospholipid concentration was approximately 13 mM in both cases.

**Figure 1:**
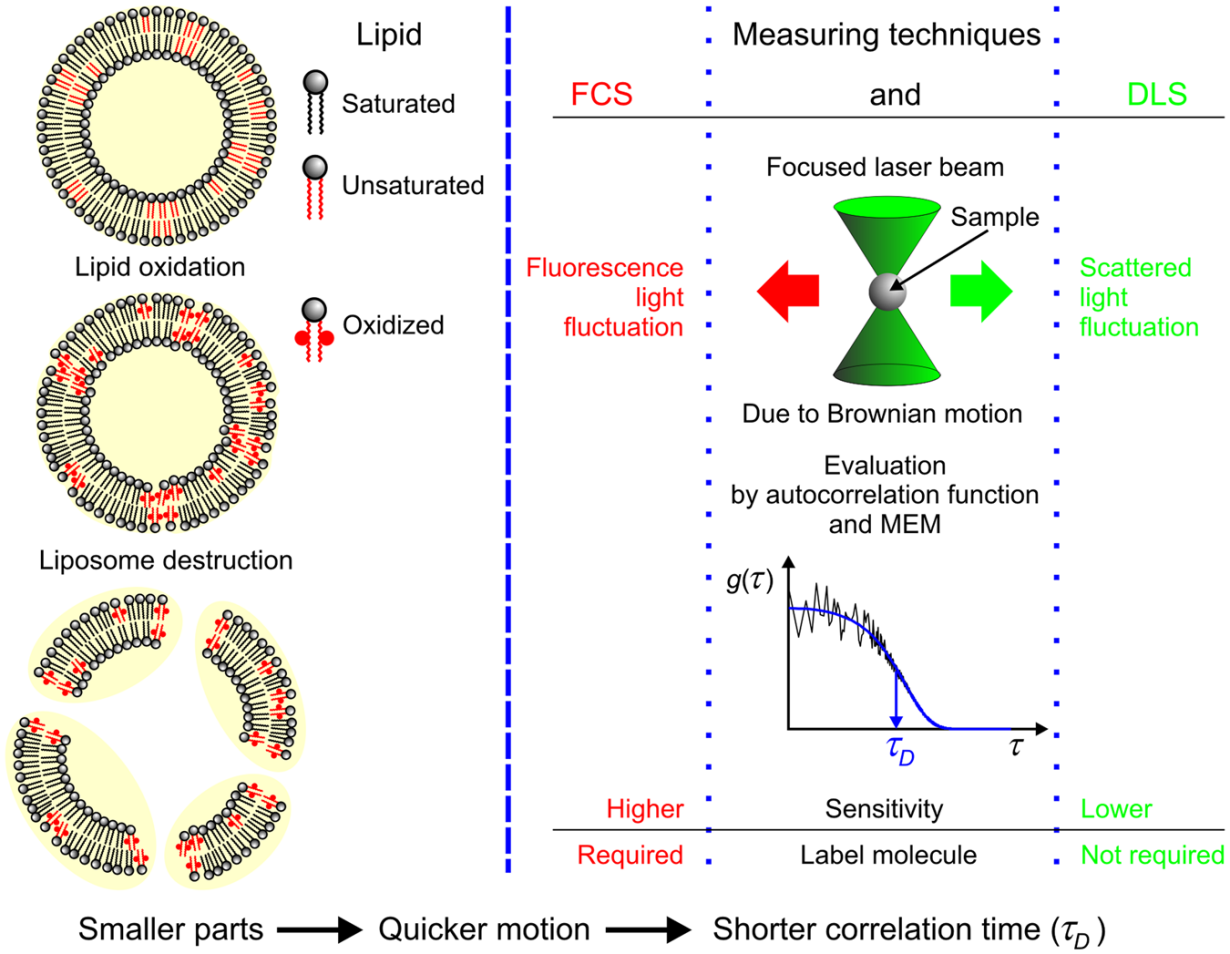
Summary of the basic experimental techniques and demonstration of disintegration of liposomes, due to oxidation of unsaturated lipids: FCS (fluorescence correlation spectroscopy) and DLS (dynamic light scattering); (MEM is the abbreviation of maximum entropy method).

For the verification of membrane disruption, we followed the change of the size-dependent Brownian mobility of the SUVs by DLS and FCS methods (see Fig. 1). For the DLS measurements it was not necessary to prepare a special sample but in the case of FCS it was. One may think that the MP which was already incorporated into the membranes as PS could be used as tracer molecule as well, but it seemed to be a better idea if the two processes were separated. Thus, it was more reasonable to use a second marker beside the MP, namely a lipophilic tracer DiO. This dye is a weak fluorescent in water but highly fluorescent and quite photostable when it is incorporated into membranes. Its excitation and emission maximum are at 489 nm and 505 nm respectively, which means that the excitation range of this dye can be found outside of the main absorption bands of MP. These properties make DiO an adequate marker for our FCS measuring conditions. DiO was added to the samples during the preparation of the lipid films. Other steps of the sample preparation were the same as described earlier. Finally, the concentration of DiO in the solution was approximately 2 μM^21^. Porphyrin was added to the liposomes at room temperature (RT ≈ 22 °C), significantly below the saturation concentration (<40 μM) at any cases, since we wanted to minimize the amount of free MP in the samples^16^.

### White light irradiation and absorption spectrometry

In order to produce ROS, we irradiated our samples in 1, 2, or 5 min steps, according to the quicker or slower change of triiodide absorption, with a Fibrolux halogen tungsten lamp (Fibrolux GmbH., Wallau, Germany). First, we measured the absorption spectra of triiodide after each step in the whole 250–450 nm range, but later just at the maximum, in the range of 350–360 nm and calculated the mean absorbance. For these measurements, we made a special optical upgrade for our Cary 4E UV-VIS absorption spectrophotometer (Varian, Inc., Palo Alto, California). With the help of this self-built optical setup, the Fibrolux light source was able to irradiate the samples through an optical fiber, and just after the irradiation a prompt measurement was done. The final illuminance of the samples was about 500 klux.

### Dynamic light scattering (DLS)

DLS equipment is composed of a goniometer system (ALV GmbH, Langen, Germany), a diode-pumped solid-state (DPSS) laser light source (Melles Griot 58-BLS-301, 457 nm, 150 mW), and a light detector (Hamamatsu H7155 PMT module). We measured the change of the scattered light intensity with a high time resolution (<5 µs), in a rather small volume (<1 nl) of the samples. Autocorrelation was used to process the fluctuation of the scattered light intensity. The autocorrelation functions were analyzed by the maximum entropy method (MEM), which enables the determination of the diffusion coefficient of the vesicles^22^. For spherical particles, this coefficient is proportional to the weight of them. This proportionality holds a squared relation with the radius (*r*) of the liposomes. Finally, with the weighting factor, *r*^−2^ the relative frequency distribution of the hydrodynamic radius was determined.

### Fluorescence correlation spectroscopy (FCS)

The home-built FCS instrument is based on an Olympus IX70 inverted microscope (Olympus Optical, Hamburg, Germany) with an UplanApo/IR 60× water immersion objective lens with a numerical aperture (NA) of 1.2^23,24^. A compact module was constructed and attached to the video port consisting of confocal optical paths for one excitation and two detection channels, and provides a diffraction-limited focus. For excitation, we used a 491 nm diode laser (Cobolt, Stockholm, Sweden) coupled via a monomode fiber into the module and with a dichroic mirror (505DRLP02) through a filter (488DF22) into the microscope. For the detection of fluorescence, we used a bandpass filter (535DF35) (all from Omega Optical, Brattleboro, VT) and an actively quenched avalanche photodiode (SPCM-AQR-13, Perkin-Elmer, Wellesley, USA), behind a pinhole with a diameter of 100 μm. The signals coming from the APDs were fed into an ALV-5000/E correlator card (ALV Laser GmbH, Langen, Germany), where intensity fluctuations were recorded and their autocorrelation function simultaneously, almost in real-time, calculated. The autocorrelation functions were analyzed by the same method as in the case of DLS^22^. The laser intensity was adjusted (≈10 kW/cm^2^) with the help of a polychromatic acousto optical modulator AOTF Nc (AA Opto Electronic, France) and measured in the focus with a Nova Display power meter (Ophir Optronics, Jerusalem, Israel). Sample solutions were measured in chambered coverglasses with a thickness of 0.14 mm (Nalge Nunc, Naperville, IL). All FCS measurements were taken in a sample volume of 0.4 ml. Applying this measuring setup, temporal fluctuations in the fluorescence emission provide information about the Brownian motions occurring on the microsecond to second time scale.

## Results

### ROS forming ability of MPs in different liposomes due to white light irradiation

**Figure 2:**
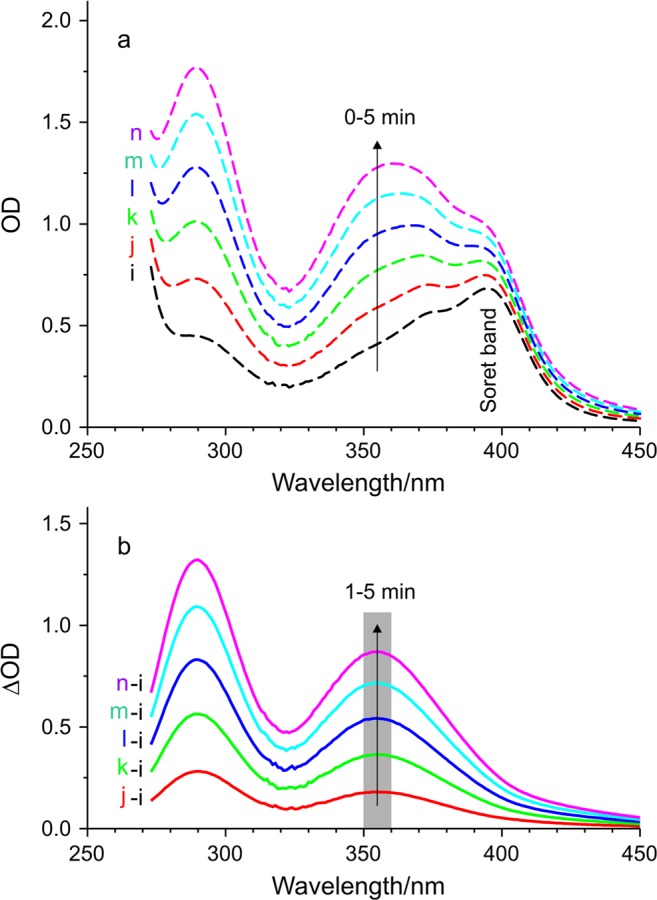
Light-induced ROS formation indicated through triiodide production followed by absorption spectrometry. (**a**) A typical series of absorption spectra of MPCl + liposome sample during the photoreaction with increasing optical density (OD) due to increasing irradiation time (0–5 min). (**b**) Difference of the two respective absorption spectrum, namely the actual minus the initial one. Gray part marks the averaging range for Fig. 3, arrow shows the increasing irradiation time (1–5 min; irradiance: 500 klux).

Figure 2a shows a typical series of absorption spectra of MPCl + liposome sample during triiodide (I_3_^−^) production according to Eq. (1), due to white light irradiation (illuminance: 500 klux, 0–5 min). It is observable that the Soret band of MPCl at around 400 nm is superimposed to the spectrum of I_3_^−^. For a better evaluation, the initial spectrum (marked with i in Fig. 2a) was subtracted from each and we got a series of difference spectra which are presented in Fig. 2b. As it is observable, these spectra consist of two well-separated bands. While in the narrower band, at shorter wavelengths the change of optical density (OD) is more pronounced, we used the broader band with a maximum at about 355 nm for further analysis in all cases, according to the original assay^17^. We chose the 350–360 nm wavelength range for the averaging of the OD and based on Beer’s law together with Eq. (1) these values are suitable for characterizing the amount of ROS. It is also observable that there is no change in the Soret band intensity, meaning that the amount of MPCl is not affected by the irradiation.

**Figure 3:**
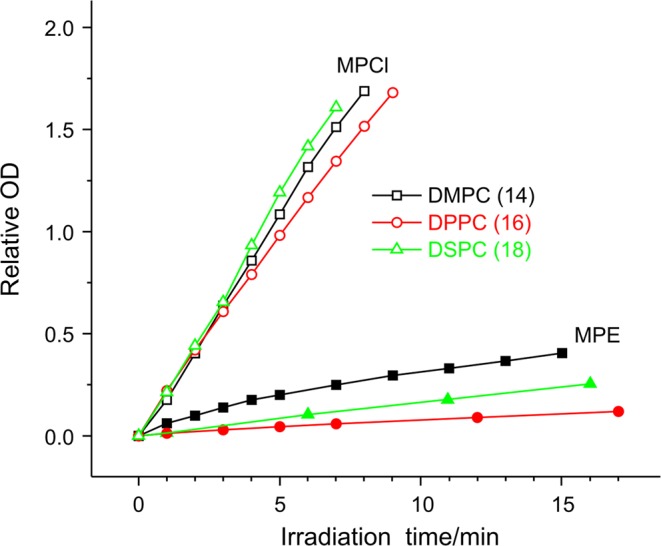
Relative mean optical density (OD) in the 350–360 nm range of the two series of MP + liposome samples during the triiodide production as a function of irradiation time. Open symbols mean data for MPCl, closed symbols data for MPE. The various carbon chain length of phosphatidylcholines as membrane components is marked with colored symbols (black square 14, red circle 16, green triangle 18), (irradiance: 500 klux).

From Fig. 2b we can derive the kinetic curves seen in Fig. 3. These curves show the mean OD of MPCl + liposome sample close to the maximum of the band as a function of the irradiation time. The mean OD is proportional to the amount of I_3_ˉ and thus to the amount of ROS. Consequently, we can follow the kinetic of ROS formation in the different samples. As it is observable, the presented part of the curves seems nearly like straight lines; therefore, we may use their slope for characterizing the ROS forming ability of MPCl and MPE in different membrane environments (relative slopes are given in Table 1).

**Table 1:** Relative slopes of kinetic curves from Fig. 3, and for DPPC + DOPC sample.

	DMPC	DPPC	DSPC	DPPC_80_ + DOPC_20_
MPCl	3.54 ± 0.08	3.38 ± 0.07	3.83 ± 0.05	9.170.02 ± 0.03
MPE	0.74 ± 0.03	0.13 ± 0.02	0.28 ± 0.02	0.310.02 ± 0.03

It is conspicuous that in general, MPCl can form the ROS with much higher efficiency than MPE can. If we consider the relative slopes of the kinetic curves in Table 1, we may state that the ROS forming ability of the MPCl is on average about ten times greater than that of the MPE. It is interesting to compare the ROS forming ability and the binding ability of MPCl and MPE in the different SUVs. While MPCl has more pronounced ROS formation than MPE, it has a moderate binding and vice versa. It should also be noted that separately for MPCl and MPE the order of slopes in Table 1 (their dependency on hydrocarbon chain length) corresponds to the order of the binding constants (*K*_b_), determined earlier^16^. Correlation coefficients of the two data series are r = 0.95 for MPCl and r = 0.89 for MPE.

### Seeking direct evidence for membrane destruction

Since we realized that the ROS forming ability of the MPCl is much more efficient than that of the MPE, we prepared samples with this PS to demonstrate the liposome disruptions due to photoreaction. DLS seemed to be a plausible technique for this purpose because the size distribution of SUVs provides a good reference about their extent. Although, we have known that in principle ROS cannot interact with saturated lipids, we tested it to be used as a negative control. The most frequently used sample was the DPPC + MPCl, but we also prepared samples with other saturated lipids. We repeated the experiment several times but significant changes in the size distribution functions were not observable.

At this point, we prepared a modified sample, containing unsaturated phosphatidylcholines as membrane components. The new membrane model consisted of a mixture of DPPC and DOPC in 80%/20% (m/m) ratio and MPCl as PS because the ROS forming ability of it in the mixed lipid sample was also much higher than that of the MPE (see Table 1). However, we also got negative results as we could not observe detectable light-induced changes in the size distribution.

**Figure 4:**
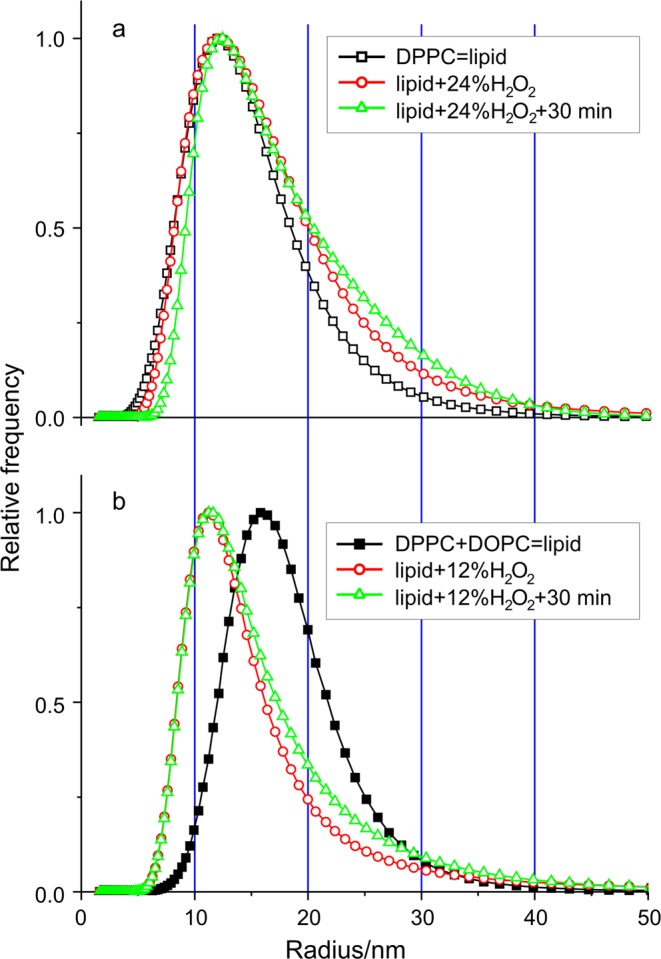
Verification of the membrane disrupting effect of H_2_O_2_ as ROS is added to two different combinations of liposome samples measured by dynamic light scattering (DLS). (**a**) Relative frequency distributions of the liposome radius of monocomponent DPPC sample without and with 24% (m/m) H_2_O_2_ added furthermore the distribution in 30 min after the ROS addition. Curves are normalized to the height for easier comparison. (**b**) The same kind of distributions of DPPC_80_ + DOPC_20_ sample without and with 12% (m/m) H_2_O_2_ added furthermore the distribution in 30 min after the ROS addition. (SUV preparation method 1).

Afterward, we modified our concept and tried to produce a disrupting effect with the direct addition of H_2_O_2_ as ROS. First, we added H_2_O_2_ in 12% (m/m) concentration to both types of SUVs and compared the membrane disrupting capabilities. Since in the case of DPPC SUV sample it did not cause any change, we doubled the amount of ROS, to 24% (m/m). In the latter case, we observed some changes, but they did not show indication of SUV disruption at all as they did not show signs of disintegration. As it is visible in Fig. 4a, the mode (most frequent value) of size distribution did not change, only the ratio of bigger to smaller sizes increased a bit and the whole distribution broadened, but smaller particles were not formed. These changes became more pronounced after a 30 min waiting time, due to presumable aggregation of the liposomes.

Contrarily, in the case of DPPC_80_ + DOPC_20_ sample after 12% (m/m) H_2_O_2_ addition, we could observe the expected changes that is the appearance of smaller particles in the size distribution, which refer to disruption. Figure 4b shows the result of this effect, namely the size distribution of the mixed sample made from saturated and unsaturated phosphatidylcholines. The 30 min waiting time after the ROS addition caused similar changes to the case of the saturated sample: a broadening towards the bigger sizes.

### Photoinduced disruption of mixed SUVs

**Figure 5:**
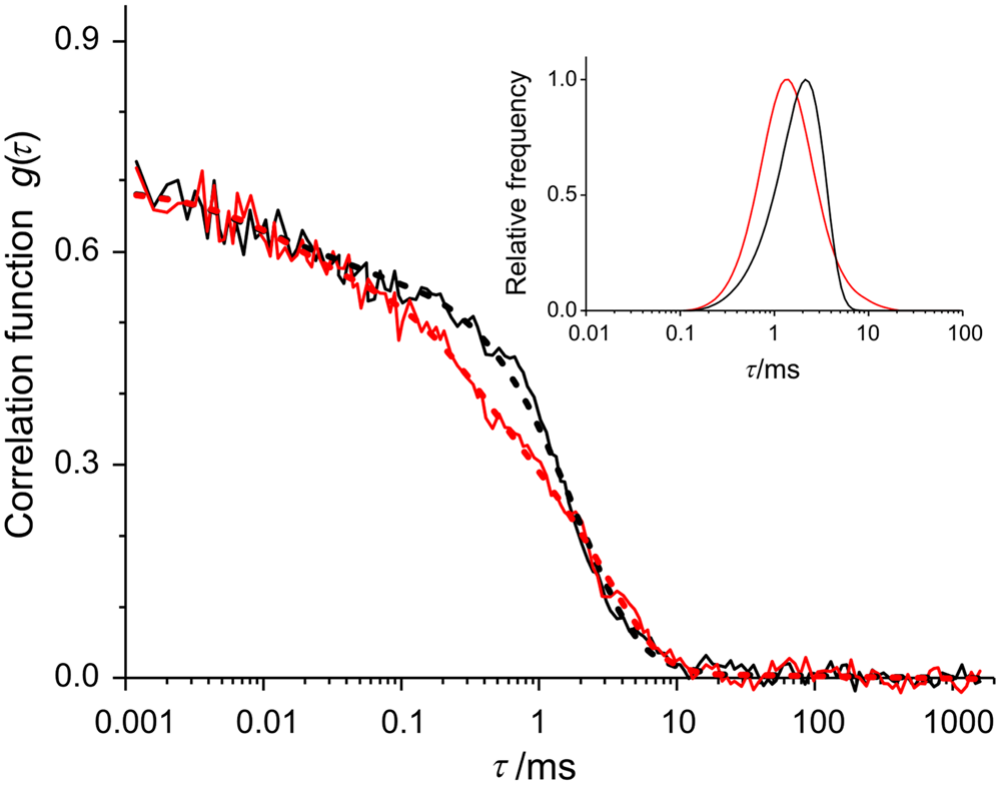
Evidence for liposome membrane disrupting effect of irradiation due to ROS formation. Results of fluorescence correlation spectroscopy (FCS) measurements of DPPC_80_ + DOPC_20_ + MPCl + DiO sample. Change of autocorrelation function due to 15 min white light irradiation (500 klux) (before: black, after: red; measured: continues line, fitted: dashed line). Inset shows the relative frequency distributions of correlation time (*τ*) based on the evaluation of the two autocorrelation function prepared by maximum entropy method (MEM). Curves are normalized to the height for easier comparison.

After observing the existence of the membrane disruption, we tried to induce the same effect by white light irradiation of the photosensitized liposomes. We used the same membrane components (DPPC_80_ + DOPC_20_) and MPCl as earlier, but because of the negative results observed in DLS, we turned to a more sensitive method, namely fluorescence correlation spectroscopy (FCS). As it was described in the Methods section, besides the MP we applied another dye (DiO) for the FCS technique. Thus, we could separate the process of the photoreaction and the phenomenon of fluorescence. Figure 5 shows the two autocorrelation functions of the mixed liposomes before and after the white light irradiation. Based on these data the relative frequency distributions of the correlation time (*τ*) was determined by the maximum entropy method (MEM). We got very similar results in their nature that is visible in the inset of Fig. 5, to that of the direct ROS addition in the DLS experiment, seen in Fig. 4b. The mode (most frequent value) decreased from 2 ms to 1.4 ms after irradiation, but the average remained practically unchanged (2.3 ms before, 2.4 ms after) and a small broadening is noticeable. Since the shorter correlation time corresponds to smaller particles, the observed changes could be considered as a consequence of the disruption of liposomes. The only difference compared to the direct ROS (H_2_O_2_) addition experiment was that in this case, the ROS is formed because of the white light irradiation in the same mixed SUV (DPPC_80_ + DOPC_20_), containing MPCl and DiO.

### A further check of disruption

**Figure 6:**
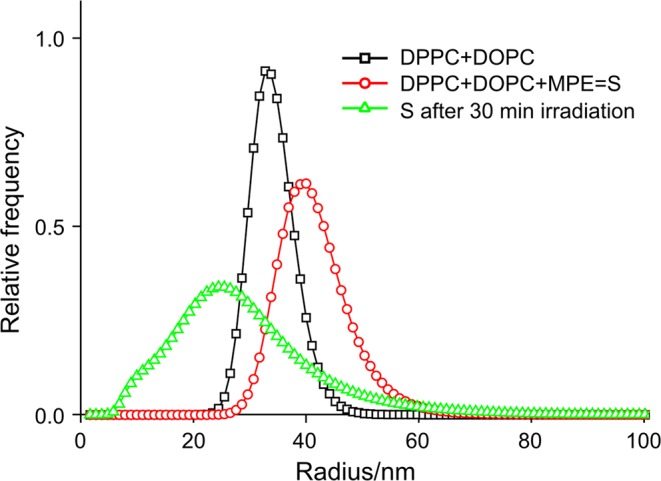
Liposome membrane disrupting effect of white light irradiation due to ROS formation on DPPC_70_ + DOPC_30_ + MPE sample measured by dynamic light scattering (DLS). Relative frequency distributions of the liposome radius of the sample: just after the preparation (black squares); after the MPE addition (red circles) and after 30 min irradiation (500 klux) (green triangles). Here the curves are normalized to the area. (SUV preparation method 2).

In the FCS experiments, we managed to present the membrane disrupting effect due to white light irradiation, but a vital question arose in connection with the particular sample; namely the presence of DiO as a new component of the SUV. We thought that this component might falsify our results. Therefore, we needed some disproof which can exclude the possibility of artifacts because we wanted to be sure that the disruption effect occurs in similar systems without the application of DiO. Thus, we used the DLS technique again with a more sensitive sample, namely with more unsaturated lipid content, but the PS was the previously used MPCl. When this experiment showed no significant changes, for contrast, we applied the more hydrophobic MPE as PS in the sample. Figure 6 shows the relative frequency distributions of the liposome radius of the DPPC_70_ + DOPC_30_ sample, without and with MPE and after white light irradiation. The effect is conspicuous and seemingly surprising. Despite the much less efficient ROS forming ability of the MPE, we could clearly verify the SUV’s disruption in such a system.

## Discussion

After determining the ROS forming ability of the two MP in several SUV samples, we looked more closely at the problem of photodamage of membranes. Since damage is a general term and the potential damages are multitudinous, we should start our discussion with one of its possible consequences, which is the leakage of ions and molecules through the membrane. It is known that depolarization of the electric potential on cell-membranes can be observed in cellular photosensitization. There are several papers where the researchers reported the kinetics of photosensitized dissipation of K^+^-diffusion electric potential that was generated across the membranes. Lipid oxidation can cause free passage of molecules through the membrane, and the effect of the liposomes’ lipid composition on the kinetics of the leakage of some fluorescent dyes was also studied^2,4,11,20^.

From our point of view, one of the significant results of these studies is that as the degree of fatty acid unsaturation increased, the photosensitized passage of these molecules through the lipid bilayer also increased. Contrarily, in another paper the authors presented a differing result based on decomposition rate constants where the unsaturated fatty acid was decomposed through an oxidation reaction with singlet oxygen. The decomposition rate constantly increases with the unsaturated degree, except for oleic acid. This result indicates that oleic acid is readily degraded despite its lower unsaturated degree^9^. This is the reason why we used DPPC-DOPC sample for our measurements. We assumed that this sample should be sensitive enough for our purpose.

The other important result of the studies, mentioned above is that the leakage can compromise the membrane’s integrity even before the massive mechanical destruction of the membrane occurs. We also learned that besides the photodamage, there is another possible origin of leakage through the membrane such as the fatty acid composition. The different fatty acids alter the membrane integrity of the liposomes. Using molecular dynamics (MD) simulations on model DOPC bilayers, authors showed that palmitate, a saturated fatty acid, caused greater destabilization of the liposomes (more “leaky”) than oleate, an unsaturated fatty acid^10^. For that reasons, leakage is not sufficient evidence for destruction.

If we compare the result of SUV’s disruption (see Figs 4b, 5 inset and 6) with a similar one from literature, we can get confirmation for our findings. Let us consider the size distribution of mixed liposomes post-inserted with protoporphyrin-lipid (PL-C17) before and after light irradiation^14^. The authors stated that the average diameter of the liposomes measured by DLS significantly increased. They also noted that the encapsulating fluorescent dye is leaked from the liposomes. According to their explanation these results suggest that the aggregation of the liposomes and the leakage of dye are caused by disruption of the liposomal membrane. As we mentioned earlier simply these phenomena do not provide sufficient evidence for disruption, but there is a relevant viewpoint which was not taken into consideration during their evaluation: the authors did not do any correction as we did and described in Methods section. The measured scattered light intensity is proportional to the weight of the particle, thus we have to divide the intensity by *d*^2^, where *d* is the diameter of the vesicle. Based on the presented data we executed the correction on their data and found that the new and real size distribution had smaller mode after light irradiation (89 nm before, 76 nm after). Broadening towards the bigger sizes was also observable and the average (106 nm) practically did not change. Therefore, we may declare the fact of disruption in their case as well because of the appearance of smaller particles in the size distribution. The original reason presumably is the oxidation of unsaturated membrane components induced by molecular oxygen under light irradiation.

At the end of this section, we seek to solve the seeming contradiction, mentioned at the end of the Introduction and the Result section. Namely that despite the much less efficient ROS forming ability of MPE, it can induce more efficient SUV disruption. This discussion has not yet taken the location of the PS in the lipid bilayer into consideration, but as we previously learned it, it is a very important factor. Singlet oxygen lifetime (*τ*_l_) depends on the nanoenvironment where it is formed. A typical value has been reported for the liposomal environment as *τ*_l_ ≈ 3 μs^25^, but in a recent paper, the authors revealed that singlet oxygen in membranes is extremely short-lived: instead of spending microseconds in the membrane interior, it does not even survive tens of nanoseconds^26^. Any damage that the singlet oxygen may cause in the lipids can occur only while it is diffusing in the membrane. Due to the short residence time, singlet oxygen does not diffuse long enough to act in sites other than its site of generation. Thus, the photosensitization efficiency should depend on the location of the sensitizer in the membrane^6,26^. Based on this assumption, the short intermolecular distance between oleic acid and protoporphyrin IX in the liposome bilayer membrane probably causes its fast decomposition by singlet oxygen as well^9^. It also suggests that the higher lipophilicity is often correlated with increased photodynamic efficiency^6^. It means that the efficient PS has a suitable structure to penetrate deeply into lipid membranes, while other molecules bind the membrane more superficially^8^. A similar trend was observed earlier using negatively-charged porphyrins with different size alkyl groups^7^.

This concept is also supported by a recent paper where the authors described the mechanism of photoinduced membrane rupture as being dependent on type I (direct contact) reactions between the PS and the lipids^27^. These contact-dependent processes were revealed to be essential for the progress of lipid oxidation, providing a molecular-level explanation of why membrane binding and location correlates so well with the efficiency of PSs. In another very recent publication, molecular dynamics simulations revealed how oxygen molecules distribute across the lipid membrane. Both in fluid- and gel-phase DPPC, the oxygen concentration at the lipid headgroups region was slightly lower than in the aqueous phase, but most of the oxygen molecules were located in the center of the bilayer (between the two membrane leaflets), where the oxygen concentration reached a value that was one order of magnitude higher then in the aqueous phase^28^.

**Figure 7:**
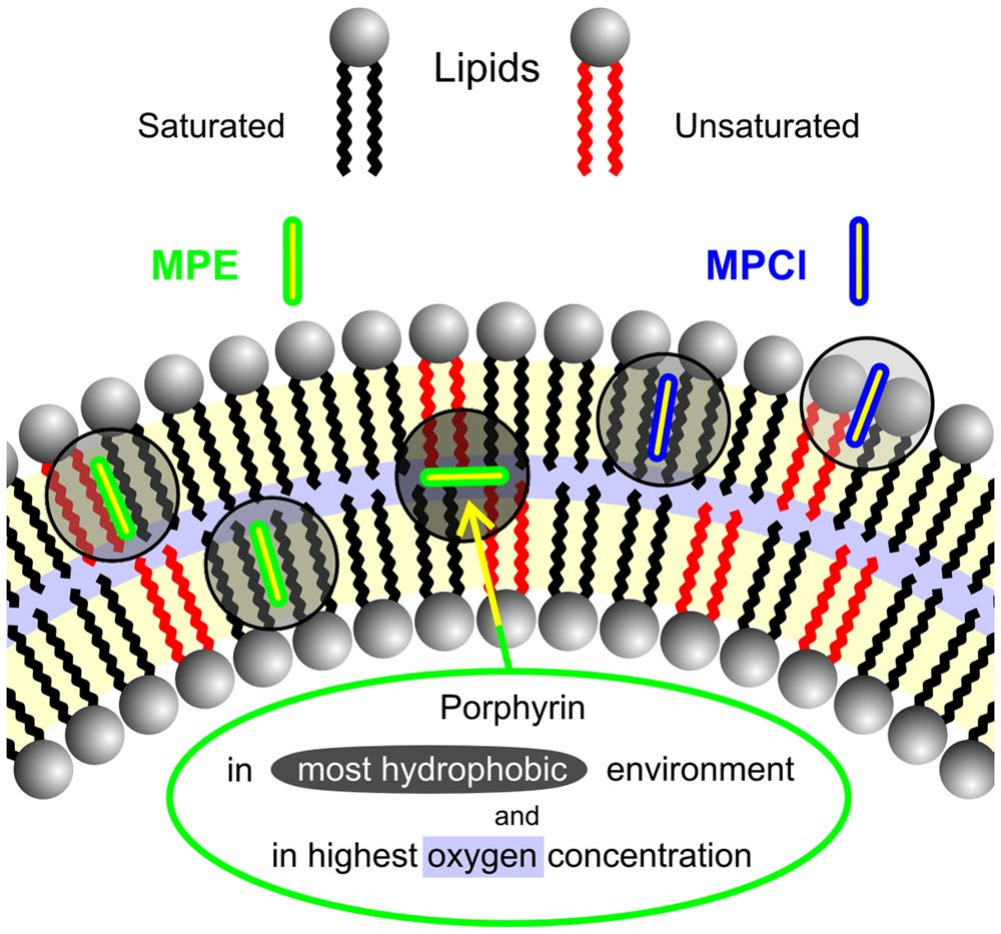
Photosensitizers lipophilicity and their location in lipid membrane are crucial in the photosensitizing effect. Though FCS and DLS techniques can not reveal the detailed molecular steps of lipid oxidation, both the contact-independent and contact-dependent pathways requires a rather short distance between the photosensitizer and the target group. Thus the most favourable location of porphyrins for high efficient photosensitizing effect is the middle of bilayer. (Increasing hydrophobic environment marked by more and more darker grey circles, the highest oxygen concentration marked by the light blue band).

According to our earlier data, MPE has about three times greater binding ability than MPCl in DPPC SUVs and it has 44% of binding the sites in between the two lipid layers (in the most hydrophobic environment, where most of the oxygen molecules accumulate) while MPCl has no such sites at all^16^ (see Fig. 7). Thus, in the light of these results, we managed to solve the seeming contradiction mentioned above.
